# Analysis and Design of Integrated Blocks for a 6.25 GHz Spacefibre PLL

**DOI:** 10.3390/s20144013

**Published:** 2020-07-19

**Authors:** Marco Mestice, Bruno Neri, Gabriele Ciarpi, Sergio Saponara

**Affiliations:** Department of Information Engineering (DII), University of Pisa; via G. Caruso 16, 56122 Pisa, Italy; bruno.neri@iet.unipi.it (B.N.); gabriele.ciarpi@ing.unipi.it (G.C.); sergio.saponara@iet.unipi.it (S.S.)

**Keywords:** rad-hard, PLL (phase-locked loop), SEE (single event effects), Spacefibre, TID (total ionization dose), charge pump, phase/frequency detector, frequency divider

## Abstract

The design of a Phase-Locked Loop (PLL) to generate the clock reference for the new Spacefibre standard is presented in this paper. Spacefibre has been recently released by the European Space Agency (ESA) and supports up to 6.25 Gbps for on-board satellite communications. Taking as a starting point a rad-hard 6.25 GHz Voltage Controlled Oscillator in 65 nm technology, this work presents the design of the key blocks for an integrated PLL: a Triple Modular Redundancy Phase/Frequency Detector, a Charge Pump, and a passive Loop Filter. The modeling activities carried out in an Advanced Design System have proven that the proposed PLL can be completely integrated on-chip, with a Loop Filter area consumption of only 6000 µm^2^ (considering the 65 nm technology). The design of active circuits has been carried out at the transistor level in a Cadence Virtuoso environment, implementing both system and layout rad-hard techniques, and different solutions are discussed in this paper. As a result, a compact (0.09 mm^2^), low power (10.24 mW), dead zone free and rad-hard PLL is obtained with a Phase Noise below −80 dBc/Hz @ 1 MHz. A preliminary block view and floor plan of the test chip is also proposed.

## 1. Introduction

Recently, the new Spacefibre standard [1] has been released by the European Space Agency (ESA). To cope with the data transfer of high bandwidth sensors, required in scientific, surveillance, and telecom satellite applications, Spacefibre supports up to 6.25 Gbps. The clock reference generator is a key block for the Spacefibre implementation. It must be hardened against SEE (Single Event Effects) [2] and TID (Total Ionization Dose) [3], it should work in a −55 to 125 °C temperature range, and it should sustain up to 6.25 GHz. Moreover, output frequency divided by 2 and by 4 should be supported. In aerospace applications, the radiation issues are mainly related to SEE because the TID levels are from several dozen to a few hundred krad, even lower if aluminum shields are used. As discussed in [4] with a 5 mm aluminum shielding, the estimated total TID received by three satellites for earth observation and environmental data collection, RazakSAT-1, SCD-2, and ALOS are 2.30 rads, 170 rads, and 24200 rads, respectively. Therefore, rad-hard techniques should be employed to mitigate SEEs, whose effect in PLLs (Phase-Locked Loops) has been widely analyzed and demonstrated [5,6]. Although several solutions have been proposed in the literature [7,8,9], the state-of-the-art rad-hard PLLs are limited to frequencies below 6 GHz in normal conditions(e.g. less than 3 GHz is achieved in [10,11,12]) but, in order to reach 6.25 GHz in all PVT (Process–Voltage–Temperature) corners and in environments pervaded by radiations, it is necessary to work at even higher frequencies in nominal conditions.

An issue that needs to be dealt with is that radiation hardness comes at the cost of power and area consumption, since redundancy techniques are usually implemented. Currently, the Triple Modular Redundancy (TMR) technique is one of the most effective techniques for digital blocks, such as the PFD (Phase Frequency Detector) [13]. It consists of triplicating a cell and adding a voter to choose between the outputs following a majority approach. For the Charge Pump (CP), instead, voltage-switching Charge Pumps (V-CP) have been demonstrated to be a good choice in terms of SEE mitigation, but they lead to a great degradation in terms of noise performance compared to CPs [14].

Among the state-of-the-art designs, the 4.8–6 GHz PLL proposed in [15] represents a good tradeoff in terms of power consumption, area, and noise performance, while really low-noise performances are achieved in [13] with a 2.2–3.2 GHz PLL.

In this paper, we present a low-power, low-area consumption and reliable 6.25 GHz PLL for aerospace environments. Here, a complete project flow in 65 nm TSMC technology is developed starting from an already designed Voltage Controlled Oscillator (VCO) [16], from an already designed Frequency Divider (FD), and from a preliminary system level analysis performed by us in the conference work [17], of which this work is an extension. With respect to [17], in which the modeling activities and the preliminary system level analysis and design were presented, in this work, beside this, the complete development at the circuit and layout level of the blocks that compose the PLL in 65 nm TSMC technology is illustrated, together with the implemented rad-hard techniques, and with comparisons among different solutions.

The Loop Filter’s components and the Charge Pump’s current have been chosen through a system-level analysis carried out in an ADS (Advanced Design System) environment, while the design of the Charge Pump and the Phase/Frequency Detector has been carried out in a *Cadence Virtuoso* environment. A preliminary block view and floor plan of the test chip is also proposed.

Hereafter, Section 2 presents the PLL system-level analysis and the design of the passive Loop Filter circuit. Section 3 deals with the transistor-level design of the main circuit blocks. Section 4 shows the results of the SEE simulation and post-layout simulations of the whole PLL. In Section 5, a starting point for a test chip is proposed, while the conclusions are drawn in Section 6.

## 2. System-Level Design of PLL and Passive Filter Sizing

**Figure 1:**
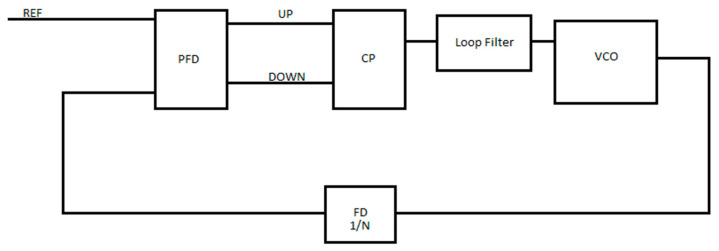
Block schematic of the Charge Pump Phase-Locked Loop (CP-PLL).

The target architecture of this work is a CP-PLL [18]. As shown in Figure 1, the blocks that compose the proposed PLL are a PFD, a CP circuit, a passive Loop Filter, a VCO, whose output corresponds to one of the PLL outputs, and an FD with an integer divide ratio, which generates the other three frequencies required by the application. The input signals of the PFD are the reference signal (REF in Figure 1) and the FD output, while the outputs related to the input phase/frequency difference are named UP and DOWN in the figure. These signals are converted by the CP in a charging or discharging current needed to control the VCO through the Loop Filter. The PLL proposed in this work is designed starting from an LC-tank VCO macrocell, which is integrated by the University of Pisa in 65 nm CMOS technology [16] and has the following characteristics: a frequency tuning range between 5.9 and 7.5 GHz, obtained through integrated varactors; the control voltage leads to a gain in the center of the frequency range of 2 GHz/V; the Phase Noise is below −100 dBc/Hz at 1 MHz from the carrier.

Starting from the system-level analysis and design, the Loop Filter’s components, the Charge Pump’s current (I_cp_), and the Frequency Divider ratio (N) have been chosen. Thanks to a divider ratio of 40 and with 156.25 MHz as the reference frequency, the output at 6.25 GHz is obtained. According to the Spacefibre standard, the PLL has also to generate the frequencies 3.125 GHz and 1.5625 GHz, which are obtained using an integer FD with the following ratios: one-half, one-fourth, and 1/40th. 

**Figure 2:**
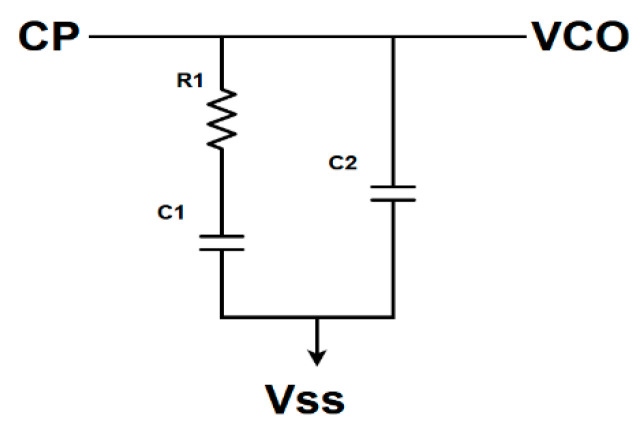
Loop Filter’s schematic view.

Moving onto the Loop Filter, as shown in Figure 2, it is composed of a capacitor C1, a resistor R1, which is needed to stabilize the loop, and a capacitor C2, whose aim is to reduce the spurious tones at multiples of the reference frequency and whose value, according to [19], should be no more than C1/5. Thanks to the modeling and simulation activity in the ADS environment in Section 2.1, a bandwidth of 6 MHz has been targeted as a tradeoff between the noise behavior and Loop Filter integrability. A completely integrated filter allows the avoidance of all the problems deriving from the parasitic effects of external components. A higher loop bandwidth, obtained with small passive devices, provides a better reduction of the VCO and Loop Filter contribution to Phase Noise. Instead, a lower bandwidth leads to lower CP and reference noise contributions, but it can be achieved only with large capacitors. A lower CP noise contribution can also be obtained thanks to a higher I_cp_, at the cost of larger capacitors for a given bandwidth. Given all these reasons, an I_cp_ of 40 µA has been chosen, and consequently, the values of C1, C2, and R1 have been derived: 8 pF, 1 pF, and 12 kΩ, respectively.

### 2.1. PLL Modeling in “Advanced Design System” Environment

A phase domain model has been firstly built to analyze the PLL bandwidth and stability. There, all the blocks are linearized: the PFD/CP and the FD have constant gains of I_cp_/2*π* and 1/*N*, respectively, while the VCO is modeled as an integrator with gain $K_{vco}$. The transfer functions for the open, Equation (1), and closed, Equation (2), loop models are the following:

(1) $$H_{ol}=\frac{I_{\mathrm{cp}}}{2\pi }Z\left(s\right)\frac{K_{vco}}{s}\frac{1}{N}\;,$$

(2) $$H_{cl}=\frac{\frac{I_{\mathrm{cp}}}{2\pi }Z\left(s\right)\frac{K_{vco}}{s}}{1+\frac{I_{\mathrm{cp}}}{2\pi }Z\left(s\right)\frac{K_{vco}}{s}\frac{1}{N}},$$

with an I_cp_ of 40 µA, *N* of 40, and:

(3) $$K_{vco}=12.57*10^{9}\;rad/\left(V*s\right),\;$$

(4) $$Z\left(s\right)=\frac{1}{s\left(C1+C2\right)}\frac{1+sR1C1}{1+sR1\frac{C1C2}{C1+C2}}.\;$$

**Figure 3:**
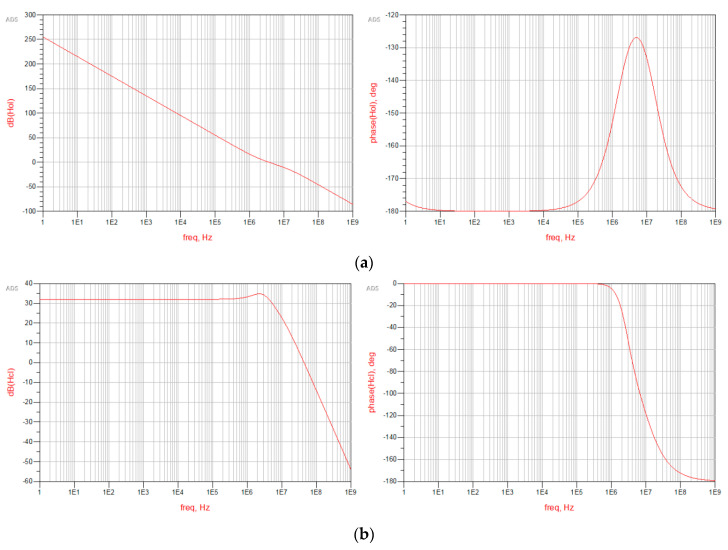
Results of the AC analysis performed on the phase domain models: magnitude on the left and phase on the right of (**a**) the open loop model and (**b**) the closed loop model.

Figure 3 shows the results obtained using an AC simulation. As expected, R1, introducing a zero, increases the stability of the loop. In contrast, C2, introducing a pole, reduces the Phase Margin; therefore, its value has been selected to maximize the latter. The main results of the simulations are a unity gain frequency of 3.31 MHz with 50.9° of Phase Margin, and a 5.37 MHz closed loop bandwidth.

**Figure 4:**
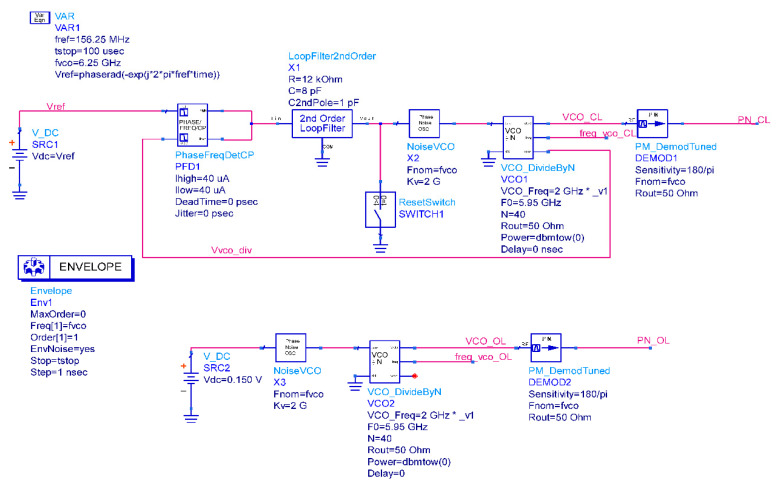
Closed-loop PLL (TOP) and Voltage Controlled Oscillator (VCO, BOTTOM) models in the time and frequency domains.

**Figure 5:**
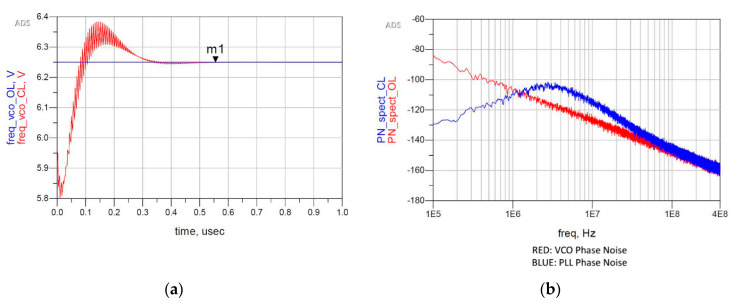
Results of the envelope analysis performed on the model of Figure 4: (**a**) locking process of the PLL; (**b**) comparison between the phase noise of the VCO and the phase noise of the closed loop PLL, considering the noise contribution of the VCO and the Loop Filter.

Secondly, to evaluate the noise and lock performances, a behavioral model of the closed loop PLL, as shown in Figure 4 together with the model of the VCO alone, has been built to perform simulations in time and frequency domains. Since all the blocks of the model are noise-free, the block labeled NoiseVCO is added to consider the VCO contribution to phase noise. It is a voltage noise source that approximates with a piecewise linear curve the simulated phase noise of the VCO designed in [16], and starting from it, it generates the equivalent input noise. Regarding the Loop Filter, the noise model provided by ADS has been used for the analysis. Figure 5a shows the PLL lock behavior, which presents a lock time of 555.6 ns for a locking error below 0.01%. The peaks in Figure 5a, due to the resistor of the Loop Filter, are not seen when the PLL is locked because the CP model is ideal. However, since the real CP will be affected by non-idealities, the second capacitor is needed to attenuate these peaks. Instead, Figure 5b shows that the phase noise is well below −80 dBc/Hz. However, the reference and CP contributions have not yet been considered in this simulation, since this represents a preliminary analysis (the PFD and CP contributions to Phase Noise are analyzed in Section 3.1.3). As expected from the theory [19], the loop has a band-pass response with respect to the Loop Filter noise and a high-pass response with respect to the VCO noise.

### 2.2. Second-Order Loop Filter’s Layout

**Figure 6:**
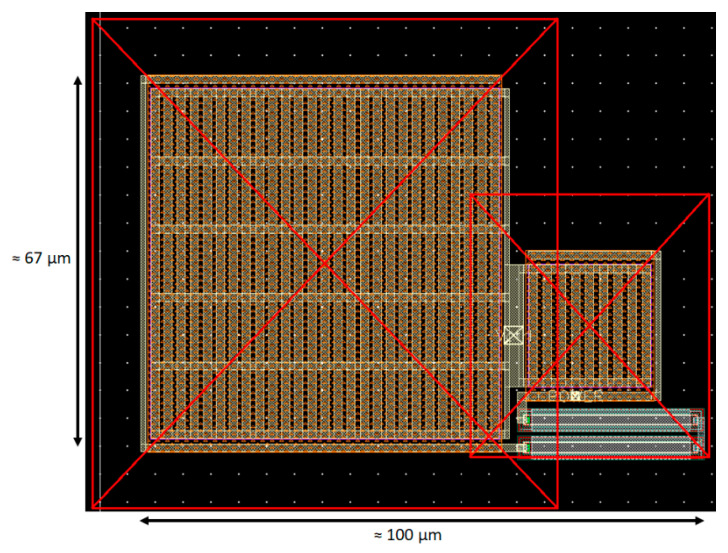
Layout in 65 nm technology of the Loop Filter in Figure 2.

Choosing to realize C1 and C2 as Metal–Insulator–Metal (MIM) capacitors and R1 as an N-well under OD resistor, in order to minimize the area consumption, have led to the Loop Filter’s layout shown in Figure 6, in which C1 can be recognized on the left, C2 can be recognized on the right, and R1 can be recognized in the lower right corner, with an area consumption of only 6000 µm^2^.

## 3. Transistor Level Design of the PLL

### 3.1. Charge Pump and Phase/Frequency Detector

#### 3.1.1. Charge Pump

Ideally, a Charge Pump is a current sink or source, depending on its inputs. To obtain this behavior, there are three main topologies of CP in the literature:

- Drain Switching,
- Source Switching,
- Gate Switching.

**Figure 7:**
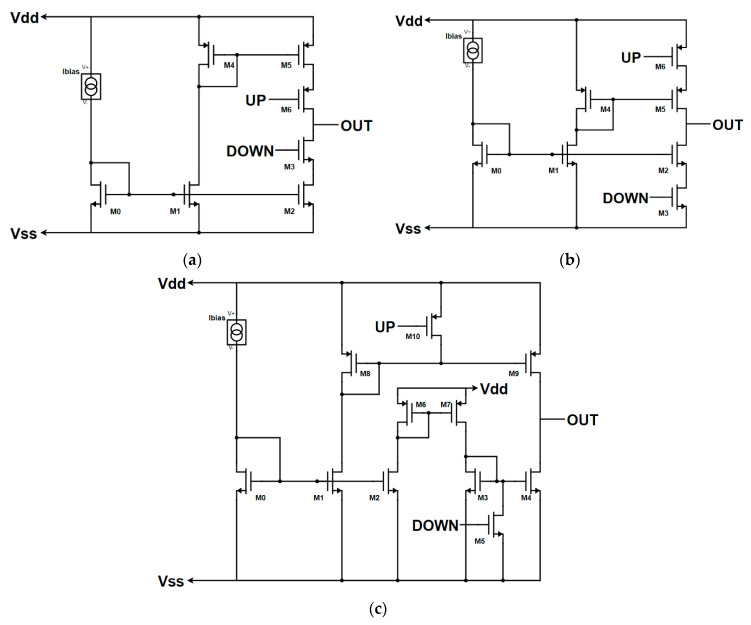
Charge Pump’s architectures: (**a**) Drain Switching architecture, (**b**) Source Switching architecture, (**c**) Gate Switching architecture.

Therefore, firstly, three basic Charge Pumps [20] have been designed and compared, one for each topology: the conventional CP’s Drain Switching architecture is shown in Figure 7a. It consists of two mirrors, one type n (M0 master) with two slaves (M1 and M2) and one type p (M4 master, M5 slave). The switch M3 is realized with an NMOS transistor, while the switch M6 is realized with a PMOS transistor. M0, M1, M2, M4, and M5 have no minimum length to obtain a higher output impedance and the necessary width to have the drain current of M3 (sink current) and M6 (source current) of 40 µA and the output voltage of 520 mV when the output is left open. 520 mV is the nominal control voltage needed to have 6.25 GHz as the output frequency of the VCO in [16]. Therefore, the CP has been sized to have near-zero current mismatch and an I_cp_ of 40 μA when the control voltage of the VCO (Vctrl) is 520 mV. Instead, the switch transistors are wide and short to minimize their V_DS_ (drain-source voltage drop).

Instead, the Source Switching-based architecture is shown in Figure 7b. Its operation is very similar to the previous CP, and therefore, it is sized with the same criteria: enhance the output impedance and reduce the current mismatch near 520 mV of Vctrl.

Finally, the Gate switching architecture is shown in Figure 7c. In contrast with the other two architectures, in this one, it is necessary to add another type p (M6, M7) and type n (M3, M4) current mirror, since causing the main type n mirror (M0, M1, M2) in the OFF state to switch off the sink current would lead to the shutdown of the source current, too. Given that, the circuit in Figure 7c has been sized with the same criteria of the other two architectures.

**Figure 8:**
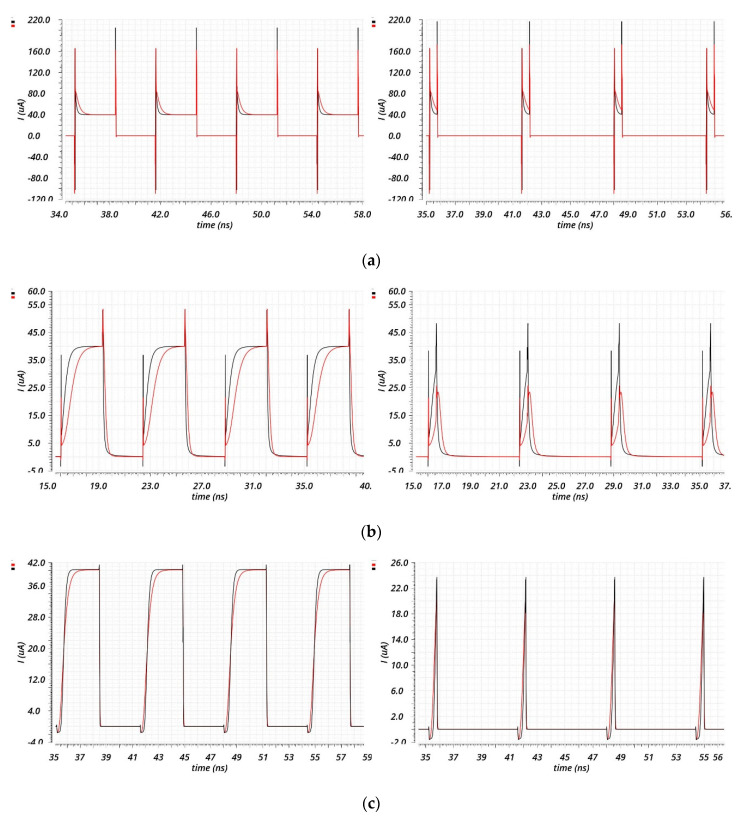
Transient behavior of the three CPs of Figure 7. The source current is represented in red, while the sink current is represented in black for two different values of ON time of the input signals (UP and DOWN): 3.2 ns on the left, which corresponds to a duty cycle of 50%, and 500 ps on the right, which corresponds to a duty cycle of 7.8125%. The Drain Switching CP results are represented in (**a**); the Source Switching CP results are represented in (**b**); the Gate Switching CP results are represented in (**c**).

To analyze the output impedance of the three CP circuits in Figure 7, imagine placing an ideal voltage source on the output node of each CP circuit. Then, we measure the output current and its derivative when UP is low and DOWN is high for the Source and Drain Switching, and when UP is high, and DOWN is low for the Gate Switching. The three CP circuits, because of their similarity, show almost the same DC characteristics: an output range between 0.15 and 0.9 V, but quite a low output impedance of about 30 kΩ. Instead, regarding the transient behavior shown in Figure 8, the three CP architectures show different characteristics. In this case, the simulations were performed forcing the output node at 520 mV with an ideal voltage source, and the source and sink currents were measured. Since the ON time of the PFD’s outputs during the reset period depends on several factors, such as the PVT conditions and layout, which were unknown at this design level, the simulations were performed considering two different values of ON time: 3.2 ns to let the two currents exhaust the transient and therefore analyze all their transient behavior; and 500 ps to approximate a more realistic reset time of the PFD and then analyze the current’s behavior in an approximation of the locked state of the PLL. The Drain Switching CP in Figure 7a is the fastest one, while the Source and Gate Switching CP ones are too slow to turn ON in less than 500 ps. The Source Switching CP in Figure 7b is the slowest one, as can be seen in Figure 8b. Both the Source and Drain Switching CP architectures show peaks during the switching period, and this leads to a degradation of the transient matching. Instead, the Gate Switching CP architecture does not show any peak current during the switching period, and therefore, although the Drain Switching is the fastest architecture and all of them can be enhanced, increasing the complexity, it has been chosen as the target topology for the whole PLL design. However, it is important to notice that this architecture is intrinsically more complex because there is the need to separate the bias circuits for the UP (M8, M9, M10) and DOWN (M3, M4, M5) branches.

**Figure 9:**
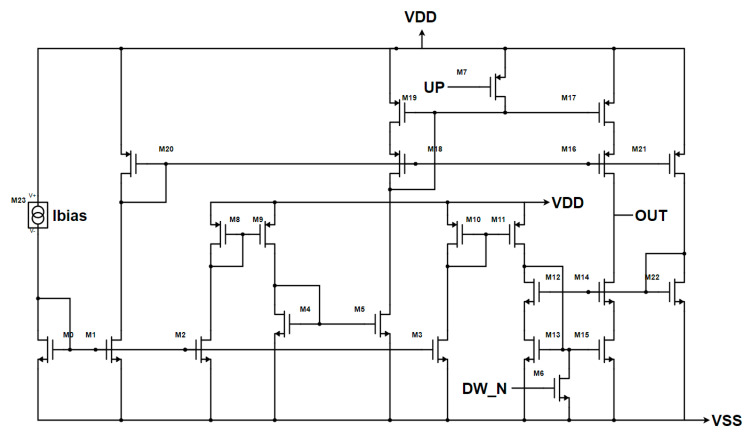
Enhanced CP’s Gate Switching architecture with CMOS standard inputs.

**Figure 10:**
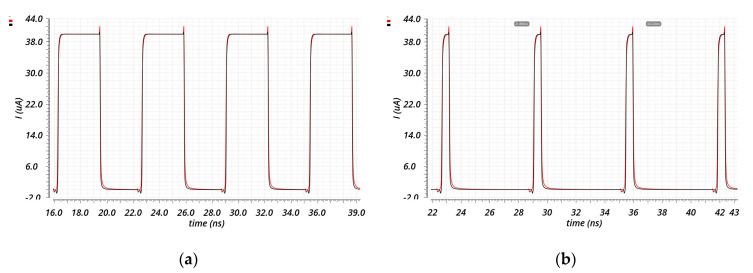
Transient behavior of the CP of Figure 9. The source current is in red, and the sink current is in black for two different values of ON time of the input signals (UP and DW_N): (**a**) 3.2 ns, (**b**) 500 ps.

Once the CP topology has been chosen, the target architecture has been modified to obtain better performances, as shown in Figure 9 versus Figure 7c. Firstly, the output simple current mirrors have been replaced with high swing current mirrors to enhance the output impedance without degrading the output range too much. This change also allowed the reduction of the sizes of the output transistors, leading to a faster CP. Moreover, both the UP and DOWN branches have their bias circuitry in order to reduce the effect of the UP signal on the sink current and of the DOWN signal on the source current. This has led to an output impedance of 250 kΩ, with an output range of 0.3–0.9 V. Instead, in terms of DC current mismatch, the worst case of process–temperature corners is 1.454 µA. Regarding the transient characteristics shown in Figure 10, the designed architecture is peak-free, and it is able to start sinking or sourcing the current in less than 500 ps.

**Figure 11:**
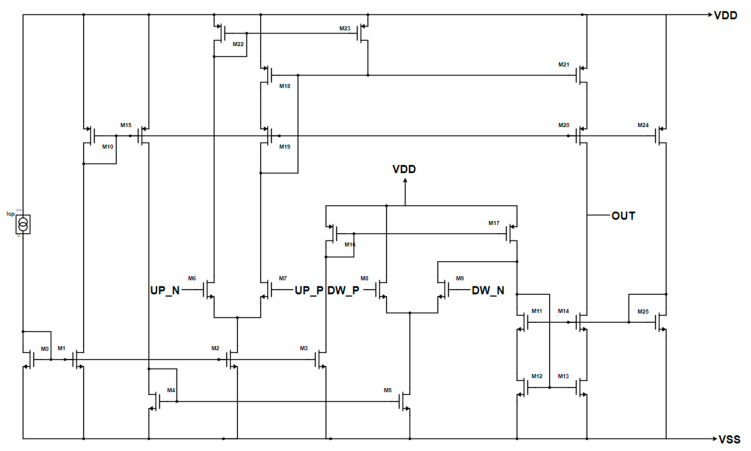
Enhanced CP’s Gate Switching architecture with differential inputs.

**Figure 12:**
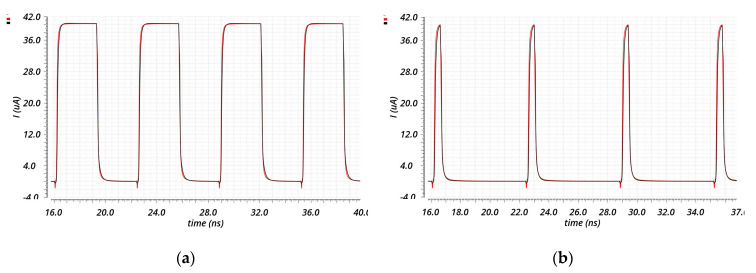
Transient behavior of the CP of Figure 11. The source current is in red and the sink current is in black for two different values of ON time of the input signals (UP_P-UP_N and DW_P-DW_N): (**a**) 3.2 ns, (**b**) 500 ps.

A differential input Gate Switching CP has also been developed. Indeed, a differential PFD/CP guarantees a better current matching during the switching period, thanks to the symmetric input load of the CP and to the lack of necessity of adding an inverter between one of the PFD outputs and the corresponding CP’s input. Hence, the switch transistors have been replaced with differential pairs (M6–M7 and M8–M9), and the mirror M22–M23 has been added to enhance the switching speed of the source current during the ON-to-OFF switch [21], as shown in Figure 11. The DC characteristics are similar to the previous architecture: an output range of about 0.3–0.9 V, an output impedance of about 250 kΩ, and a DC current mismatch in the worst case of all the temperature–process variations of 1.66 µA. Instead, regarding the transient behavior, it is peak-free and shows a better transient current matching, as can be seen from Figure 12.

#### 3.1.2. Phase/Frequency Detector

**Figure 13:**
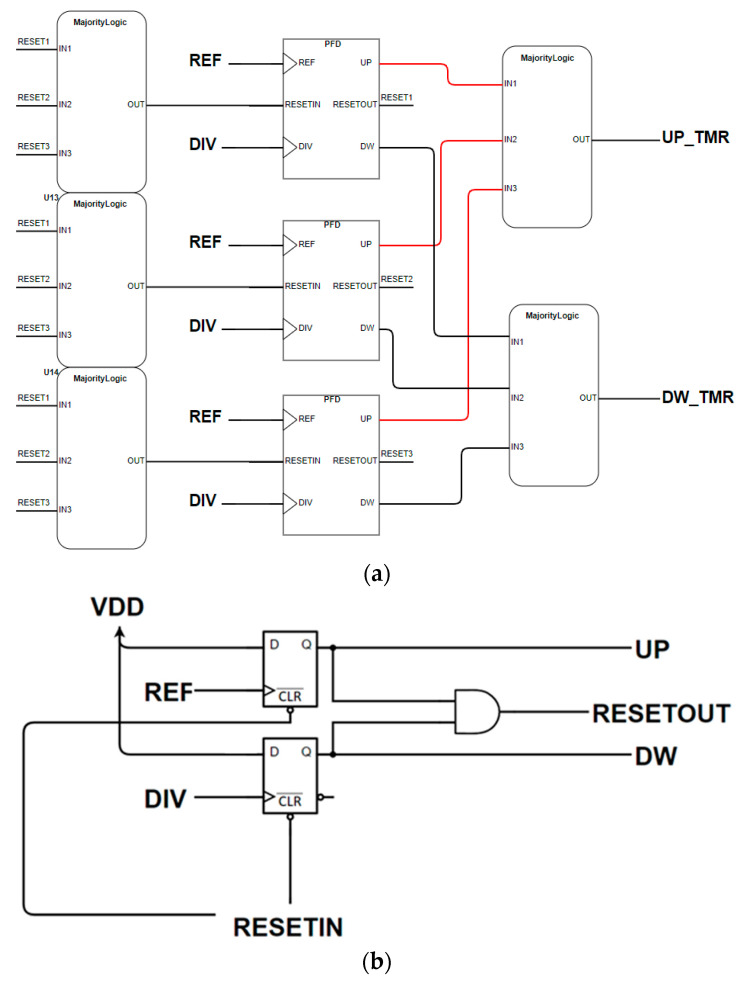
Triple modular redundant PFD architecture in (**a**) and simple PFD architecture in (**b**).

Since the PFD is essentially a digital block, the easiest and most effective way of hardening it against radiation is the TMR [13]. The designed architecture is shown in Figure 13a. The block-labeled PFDs in Figure 13a consist of the conventional PFD architecture shown in Figure 13b, and every PFD has its own reset voter that decides between all the output resets. In this way, the only possible loss of lock for the PLL happens when an SEE occurs on the UP and/or DOWN voters. However, this error does not lead the PFD into a wrong state causing cycle slipping, i.e., one of the input’s loss (or gain) of one cycle with respect to the other input. This architecture has been developed in both CMOS logic and CML (Current Mode Logic), in order to be compatible with the designed CPs.

#### 3.1.3. Comparison between the Two PFD/CP Architectures

**Figure 14:**
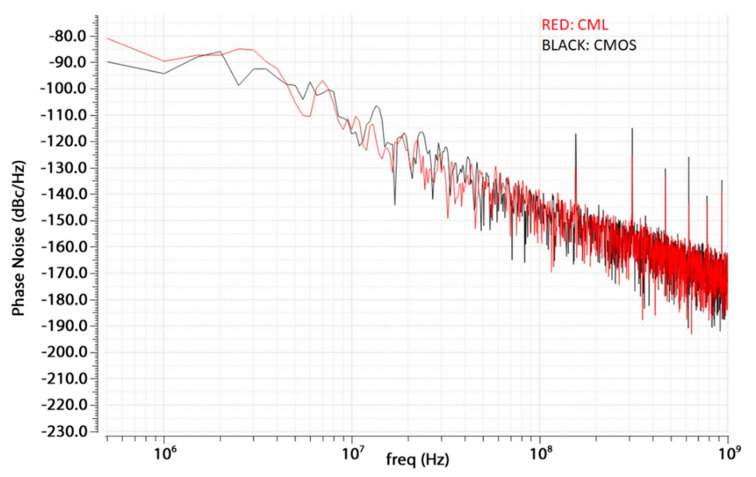
Comparison between the two CP/PFD (Phase Frequency Detector) architectures in terms of phase noise: Current Mode Logic (CML) architecture’s results in red, CMOS architecture’s results in black.

In order to compare the two different solutions (CMOS and CML versions of the PFD/CP), two PLL testbenches have been built in Cadence. In these testbenches, the VCO model with parasitic effects is considered, while the CP’s and PFD’s schematic view has been used to reduce the simulation time. Finally, a Verilog-A frequency divider, which can be found in the Cadence’s *rf-library*, has been used. For the Phase Noise comparison, because of the non-linear nature of the PLL, a direct time domain noise analysis was necessary. This has been done exploiting the Transient Noise Analysis option embedded in the Spectre RF’s Transient Analysis. Since this type of simulation is time consuming, a resolution bandwidth of 500 KHz to reduce the simulation time has been set. In Figure 14, the Phase Noise results are shown: the two solutions have almost the same behavior in terms of noise, but the CMOS solution shows higher peaks at multiples of the reference frequency because of the worse current matching during the switch of the CP currents from ON to OFF and vice versa. Instead, in Table 1, the comparison in terms of DC current matching of the CP and power consumption between the CML and CMOS solutions is summarized. The DC current mismatch has been measured performing a DC simulation on the CP circuits and measuring the output current when the output node is forced at 520 mV with an ideal voltage source, and the inputs are such that both the source and sink current are ON. Considering that (1) the noise performance difference is negligible, (2) the CMOS solution has a better current matching in the worst case, and (3) the CML solution shows 25 times higher power consumption, then the CMOS solution has been chosen and has been developed at layout level for the whole PLL design. 

#### 3.1.4. PFD/CP Layout

**Figure 15:**
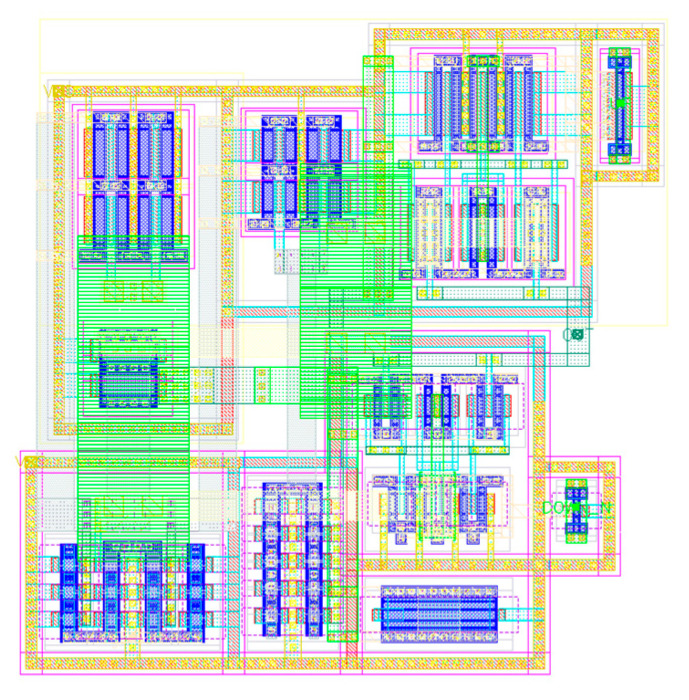
Charge Pump’s layout.

The CP’s layout is shown in Figure 15. The output mirrors, since their slaves are composed of two MOS in parallel, are interleaved with the master transistor to enhance the technology process matching. Moreover, every mirror and the switch transistors are surrounded by guard rings connected to the voltage supply. These rings have two main functions:

1. They reduce the possibility of SEL (Single Event Latch-Up) and Latch-Up in general;
2. They reduce the drift current generated after an SEE in the sensitive nodes near the hit node [22].

**Figure 16:**
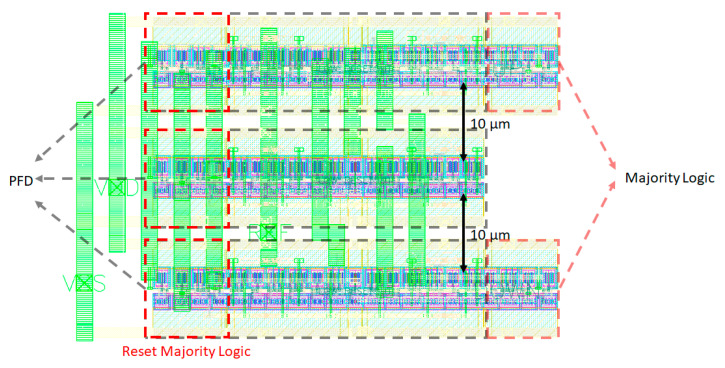
Phase/Frequency Detector’s layout.

Regarding the PFD, as for the CP, guard rings have been placed around the transistor to avoid latch-up and to reduce the drift current deriving from an SEE. Moreover, the triplicated cells have been placed at a distance of 10 µm from each other to avoid MBUs (Multi Bit Upsets). The TMR technique would become useless if an SEE causes an SEU (Single Event Upset) in more than one PFD each time. In Figure 16, the complete layout of the TMR PFD is shown: the three PFDs with their reset majority logic can be recognized on the left, while the output majority logic can be recognized on the right.

## 4. Simulation’s Results

### 4.1. PFD/CP Characterization

**Figure 17:**
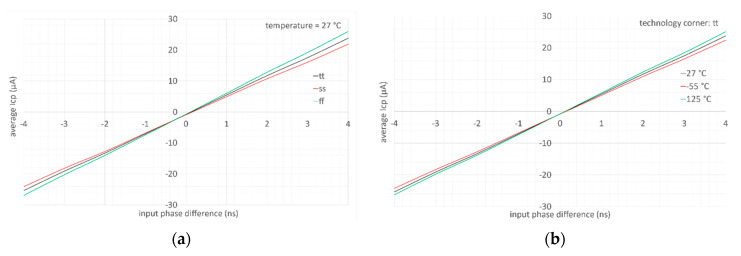
PFD/CP characteristic for different technology corners at 27 °C (**a**) and different temperatures in typical case (**b**).

In Figure 17, the average CP current is represented as function of the phase difference between the inputs for different technology corners (Figure 17a) and different temperatures (Figure 17b). As can be seen from the figure, the PFD/CP is dead zone-free thanks to the presence of the RESET state in the PFD.

### 4.2. Single Event Effect Simulations on the CP

The current caused on a sensible node by an SEE can be modeled as a double exponential function [23] in Equation (5). In this work, the parameter model of Equation (6), which was taken from a previous high-frequency design we have done in the same technology [24], has been used:

(5) $$I\left(t\right)=Q\frac{e^{-\frac{t}{\tau_{1}}}-e^{-\frac{t}{\tau_{2}}}}{\tau_{1}-\tau_{2}}\;.$$

(6) $$\tau_{1}=200÷400\;ps\;,\;\;\tau_{2}=50÷100\;ps\;,\;\;Q=67\;÷800\;fC,$$

where *τ*_1_ and *τ*_2_ are the constant times of the double exponential shape and *Q* is the charge released in the silicon substrate during a particle strike, which corresponds to a particle LET (Linear Energy Transfer) between 5 and 60 Mev∙cm^2^/mg. 

**Figure 18:**
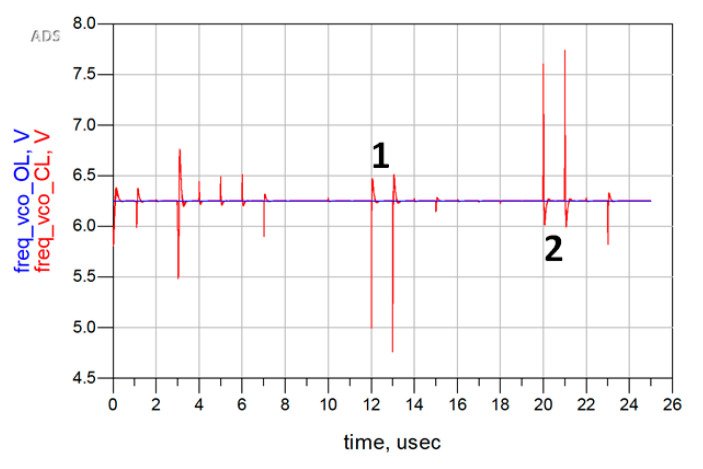
Output frequency of the ADS PLL model as function of time, for Single Event Transients (SETs) spaced 1 µs apart, hitting every sensitive node of the CP and with a Linear Energy Transfer (LET) of 60 MeV∙cm^2^/mg.

The SEE analysis is focused on the most critical block, the CP, since the PFD adopts a TMR mitigation approach, the passive Loop Filter avoids the use of feedback loops and of active circuits, while the VCO was inherited from a previous SEE-tested designed block in [16]. Hence, double exponential current generators from the Cadence’s analogLib library have been placed on every sensitive node of the CP (all the drains and sources of the MOSFETs that were not connected to the supply voltage), taking care of the direction of the current. Then these generators have been delayed to analyze their effect separately, and the output current has been measured in order to see how an SEE on each node of the CP affects the output current. Then, these results have been reported to the ADS behavioral model of the PLL to see how the PLL reacts to Single Event Transients (SETs) on the CP (see Figure 18).

**Figure 19:**
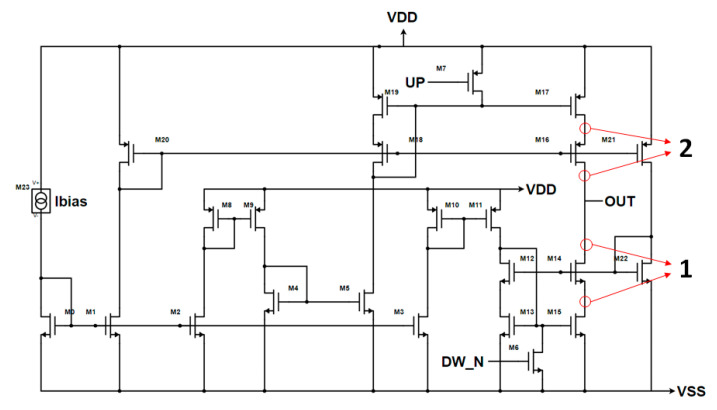
Highlight of the output nodes of the CP.

As shown in Figure 18, the PLL, which loses lock after an SET, is able to recover the locked state in less than 600 ns. The largest peaks, which are indicated in Figure 18 with the numbers 1 and 2, are the ones derived by an SET on the output nodes, as highlighted in Figure 19 with the corresponding numbers, because the charge injected into these nodes goes directly through the output node, charging or discharging the Loop Filter and then modifying the control voltage of the VCO.

### 4.3. PLL Testbench

Once all the blocks that compose the PLL have been designed, a testbench has been built. Here, the netlist generated by the layout parasitic extraction has been used for all the blocks (CP, PFD, VCO, Loop Filter, and FD), while the connections between the blocks are still ideal. On this testbench, a Transient simulation has been performed to analyze the locking behavior and the Phase Noise. As for the simulations presented in Section 3.1.3, the Transient Noise Analysis Option has been used for the Phase Noise evaluation.

#### 4.3.1. Locking Process

**Figure 20:**
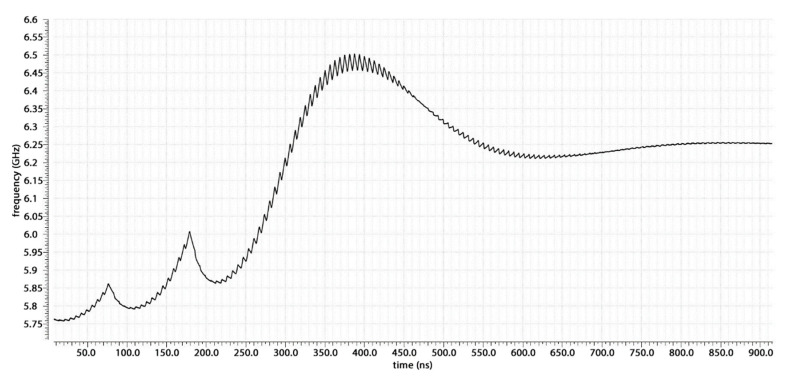
Post-layout locking process.

The locking process in the typical technology corner at 27 °C is shown in Figure 20. The lock time is 1 µs, which is twice that expected from the ADS behavioral model. This is mainly due to two factors: (1) the non-linearity of the VCO’s characteristic, and (2) the cycle slipping. In the ADS model, the VCO was approximated to have a linear characteristic with 2 GHz/V gain, but this is true only in a small range of frequencies. For the other frequencies, a smaller gain and consequently, a smaller loop bandwidth and damping factor results, which leads to a higher lock time. Moreover, as can be seen from the locking process, at the beginning of the process, two cycle slips are seen. This is due to the RESET state of the PFD necessary to cancel the dead zone. When the phase difference between the two PFD’s inputs is high enough that the new edge of the input ahead comes while the PFD is in its RESET state, this edge is not seen, and the PFD goes into the wrong state with respect to the ideal one. From that, it recovers the correct state when the cycle slip happens, but this leads to an enlargement of the lock time.

#### 4.3.2. Noise Simulations

**Figure 21:**
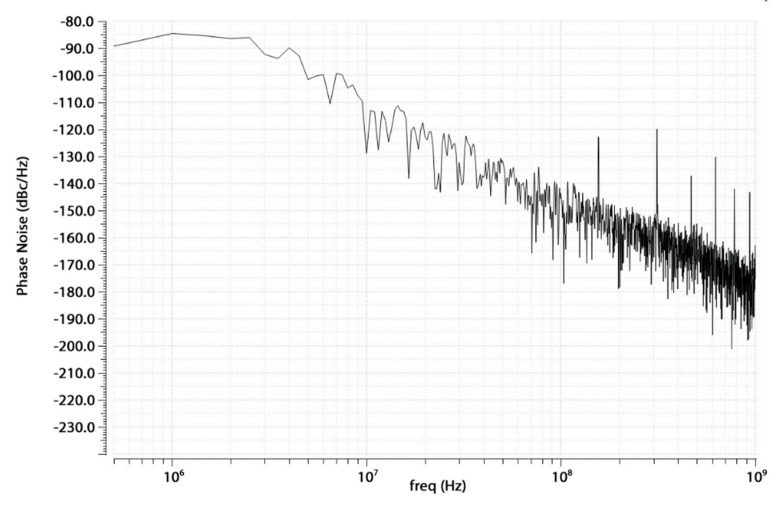
Post-layout phase noise.

In Figure 21, the resulting post-layout Phase Noise for the whole PLL is shown. It is below −85 dBc/Hz, which is in line with that of the VCO. The largest contribution is due to the CP/PFD at low and middle frequencies, while the VCO’s noise is predominant at higher frequencies, as expected from the theory [19].

Since in digital design, a temporal characterization of noise is usually preferred, the absolute Jitter has been measured, resulting in a peak-to-peak value of 8.8 ps and an RMS (root mean square) value of 2.03 ps (typical corner, considering 12,500 cycles, which in terms of Phase Noise means a bandwidth between 500 kHz and 1 GHz). Absolute Jitter is really important for communications systems, since it measures how much an edge of the clock is in a different position from the ideal one. For other applications, Period Jitter is of most interest, since it measures the difference between a clock period and the average one. Therefore, Period Jitter has been also measured, in the typical corner as well, resulting in a peak-to-peak of 96 fs and an RMS value of 14.744 fs.

## 5. Test Chip

**Figure 22:**
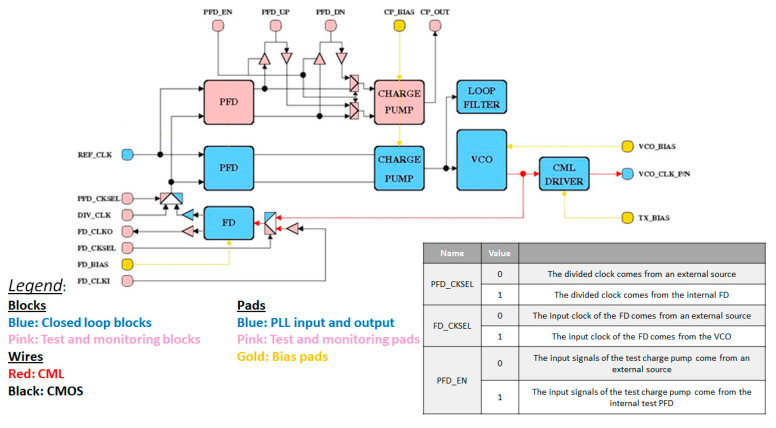
Block schematic of the test chip.

**Figure 23:**
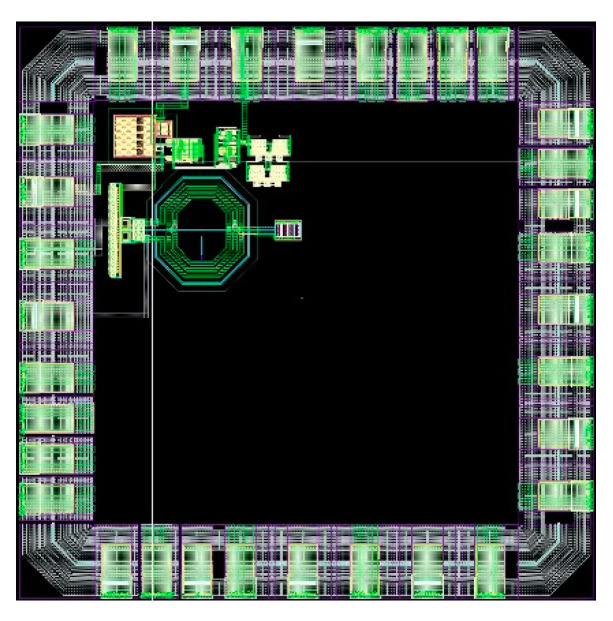
Draft floor plan of the test chip.

To prototype the designed PLL, a block view of a possible test chip has been developed and is shown in Figure 22. As can be seen, it consists of a whole PLL, which can be recognized at the bottom, and a duplicate of the PFD and CP. The main idea is to test every block separately: there is the possibility of giving the inputs from an external source, and all the outputs are outputs of the test chip. Since the PFD/CP’s area consumption is low, duplicating these blocks gives the possibility of testing them separately, without adding multiplexer and buffer inside the loop, disturbing the PLL’s operation. The FD is not duplicated because it consists of three stages that need bias currents. This means that at least 4 pads are necessary for it and, therefore, duplicating it would lead to a too high requirement in terms of pads. Moreover, in Figure 23, a draft of the floor plan is shown: the chip’s area to be fabricated using a Europractice Multi Project Wafer is 1 mm^2^. As can be seen from the figure, the whole PLL occupies an area of about 0.09 mm^2^ (the remaining area and pads are used for other designs not discussed in this paper). It is to be noted that the design of the pads is inherited from a previous design, where they were tested also with respect to resistance to radiation effects [24].

Although the figures and tables proposed in this paper refer to post-layout simulation results, the experience of our research group from previous designs in the same 65 nm TSMC technology, using the CAD (Computer-Aided Design) design environment and targeting similar operating frequencies, has proven that there is a coherent alignment between post-layout simulations and experimental measurements. In the next step, when the measures of the prototyped chip will be performed, a complete assessment of the results accuracy will be done.

## 6. Conclusions and Future Work

The design of a PLL for the new ESA Spacefibre standard has been presented in this paper. The work was carried out in ADS for the modeling activity and Cadence Virtuoso for the design activity. In particular, the design of a TMR PFD, a CP, and a passive Loop Filter has been presented, starting from an already designed 6.25 GHz rad-hard VCO in 65 nm technology. The modeling activity has shown that the PLL can be completely integrated on-chip, with a Loop Filter area consumption of only 6000 µm^2^. The PLL is able to generate three output signals at 6.25, 3.125, and 1.5625 GHz with a gain margin of 86 dB and phase margins of 50°. The Phase Noise of the PLL, considering the higher frequency output, is below −85 dBc/Hz @ 1 MHz, and hence, it is in line with that of the VCO. The PLL power consumption in the locked state is about 10.24 mW. The PFD/CP is dead-zone free and shows a current matching below 2 µA (5% of the nominal CP’s current value) in the worst case of process–temperature corners. Moreover, the PLL is highly immune to SEE on the PFD, while it is able to relock in less than 600 ns for an SEE on the CP. Finally, an area estimation has been done considering also the VCO, resulting in a total area of 0.09 mm^2^.

In Table 2, a comparison with the state-of-the-art rad-hard PLLs is performed. In [15], a CP-PLL in 65 nm technology for high-energy physics is presented. It generates a tunable output frequency in the range of 4.8–6 GHz, which is slightly lower the SpaceFibre standard. As reported in [15], the CP-PLL was not designed to be SEE tolerant; indeed, a simple PFD was used in the PLL loop. Instead, in this work, a TMR PFD was implemented to increase the hardness against SEE. In [13], all the digital logic of the CP-PLL is implemented using a TMR approach, but its low-frequency range is not suitable for SpaceFibre application. In [25], the design of a rad-hard CP-PLL that is able to generate an output frequency in the range 1.17-3.16 GHz is reported. It bases its radiation hardness on the use of a Silicon On Sapphire (SOS) substrate. Indeed, the SOS having a resistive sapphire in the substrate reduces the creation of electron–hole pairs and their migration in the device bulk in the case of energetic ion strikes. An example of radiation hardness improvement achieved using a different technology is presented also in [26], where a high-frequency CP-PLL is designed in 250 nm SiGe technology. Instead, this work presents the design of a PLL that is suitable for space applications in standard silicon CMOS technology, whose radiation hardness is achieved by TMR and layout techniques. 

The next step is test chip fabrication to prototype the proposed PLL solution. The design has been submitted to tape out through MPW (Multi Project Wafer), and hence, the experimental verification of the fabricated prototype is part of the future roadmap.

## Figures and Tables

**Table 1:** Comparison between the two CP/PFD architectures in terms of CP’s current matching and CP+PFD’s power consumption.

	CMOS	CML
Charge Pump DC current mismatch (worst case)	1.454 µA	1.66 µA
Power Consumption Charge Pump + PFD	≈200 µW	≈5 mW

**Table 2:** Comparison with the state-of-the-art Rad-Hard PLLs. SOS: Silicon On Sapphire.

	This Work	[13] *	[15] *	[25] *	[26] *
Technology	65 nm CMOS	65 nm CMOS	65 nm CMOS	250 nm SOS	250 nm SiGe
Frequency Range (GHz)	5.2–6.4	2.2–3.2	4.8–6	1.17–3.16	17.5–18.9
Power Consumption (mW)	10.24	11.7	18	102.5	-
Area (mm^2^)	0.09	-	0.124	0.52	5
Absolute Jitter (ps) (RMS)	2.03	0.345	3.23	-	-
Period Jitter (fs) (RMS)	14.74	-	3550	-	-
Phase noise @ 1MHz (dBc/Hz)	−85	-	-	−100	−110

* measured.
